# Generation of Single-Cell Suspensions from Mouse Neural Tissue

**DOI:** 10.3791/1267

**Published:** 2009-07-07

**Authors:** Sandra Pennartz, Sandy Reiss, Rebecca Biloune, Doris Hasselmann, Andreas Bosio

**Affiliations:** Miltenyi Biotec,GmbH

## Abstract

Within the nervous system, hundreds of neuronal and glial cell types have been described. Each specific cell type in the brain or spinal cord has a repertoire of cell surface molecules, or molecular determinants, through which it can be identified and characterized. Currently, robust cell identification and separation technologies require single-cell preparations to be generated while simultaneously limiting cell death and destruction of characteristic surface protein. The gentleMACS Dissociator, when used in combination with trypsin or papain-based dissociation kits, can effectively and gently dissociate brain tissue while preserving antigen epitopes and limiting cell loss. Standardized preparation of single-cell suspensions is achieved using C Tubes and optimized, preset gentleMACS Programs. Once generated, single-cell suspensions can be treated with monoclonal conjugates like Anti-Prominin-1 MicroBeads, which identify neural progenitors, or purified further using Myelin Removal Beads.

**Figure:**
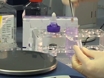


## Protocol

To work under sterile conditions, it is recommended to perform all steps in a laminar flow hood.

### 1. Materials

Neural Tissue Dissociation Kit (P) (Components: Solution 1,2 , 3, and 4, Storage Buffer)
Pre-Separation Filters
(Optional) Myelin Removal Beads
Beta-mercaptoethanol
gentleMACS Dissociator
C Tubes
MACSmix Tube Rotator
HBSS (w/o)
HBSS (w)

Prepare the following solutions prior to beginning the protocol:
  Hanks’ Buffered Salt Solution without divalent cations Ca^2+^ or Mg^2+^ (HBSS (w/o)
  HBSS, standard, i.e. with Ca^2+^ and Mg^2+^ (HBSS (w)
Depending on the antigen epitope of interest in subsequent applications, use either the Papain-based Neural Tissue Dissociation Kit (P) or the Trypsin-based Neural Tissue Dissociation Kit (T).
Prepare the following solutions from the Neural Tissue Dissociation Kit:
  Solution 2: add beta-mercaptoethanol to 0.067 mM
  Solution 4: dissolve powder in 0.7 ml Storage Buffer (provided in kit), gently mix (do not vortex)
  Enzyme Mix 1: Pipette 1.9 mL Solution 2 and 50 μl Solution 1 (both from kit) into a C Tube and incubate for 10-15 min at 37 °C. This is sufficient to dissociate 400 mg of brain tissue.

### 2. Dissociating the neural tissue

Weigh brain tissue in a tube containing 1 ml HBSS (w/o)
Transfer mouse brain into the C Tube containing 1950 μL of preheated Enzyme Mix 1. (NOTE: this protocol is written for ≤ 400 mg, but solution volumes can be scaled to accommodate up to 1600 mg of brain tissue per C Tube)
Place the C Tube onto the gentleMACS Dissociator and run program “m_brain_01”.
Incubate with rotation (4 rpm using a MACSmix Tube Rotator) for 15 min at 37 °C.
Place the C Tube onto the gentleMACS Dissociator and run program “m_brain_02”.
During the dissociation step prepare 30 μl of Enzyme Mix 2 per 400 mg of tissue by gently mixing 20 μl of Solution 3 and 10 μl of Solution 4.
Add Enzyme Mix 2 to the C Tube via the septum-sealed cap and mix gently without vortexing.
Incubate the C Tube with rotation for 10 min at 37 °C in an incubator.
Place the C Tube onto the gentleMACS Dissociator and run program “m_brain_03”.
Incubate the C Tube with rotation for 10 min at 37 °C in an incubator.
Centrifuge briefly to collect the sample at the bottom of the tube.

### 3. Filtration

Select a filter large enough to allow passage to the cells of interest. For instance, Purkinje cells are too large to pass through a 30 μm filter, but Prominin-1+ cells will.
Use a suitable1000 μl pipette to remove the cells form the C Tube through the septum-sealed cap and apply it to the Pre-Separation Filter on a 15 ml collection tube. (NOTE: for larger sample sizes use a 50 ml collection tube).
Wash the filter with 10 ml of HBSS (w).
Centrifuge the filtered cells at 300 x g for 10 min at RT.
Resuspend the supernatent in your medium or buffer of choice for subsequent applications.
To remove myelin debris, use Myelin Removal Beads.

### 4. Representative Result: Please See Figures 1-3


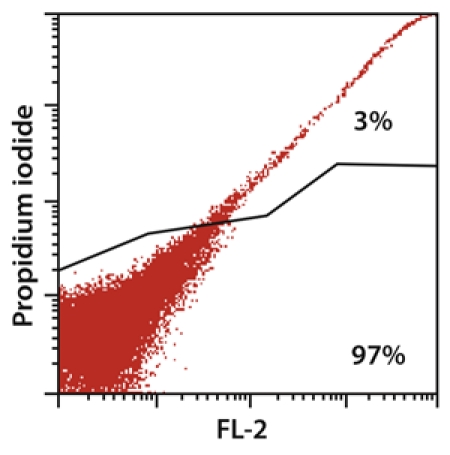
 **Figure. 1** The brain dissociation with the gentleMACS Dissociator resulted in 97% viable cells, as flow cytometric analysis shows.


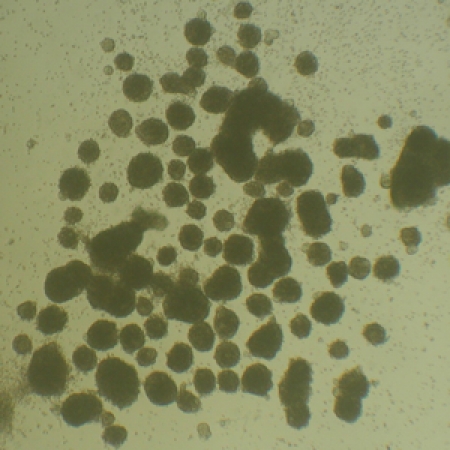
 **Figure. 2** Light microscope picture of neurosphere formation after magnetic cell sorting using Anti-Prominin-1 MicroBeads after 7 days of cultivation in MACS® NeuroMedium supplemented with MACS Supplement B27 PLUS. Cells were prepared from CD1 mouse brain (P3) using the Neural Tissue Dissociation Kit (P).


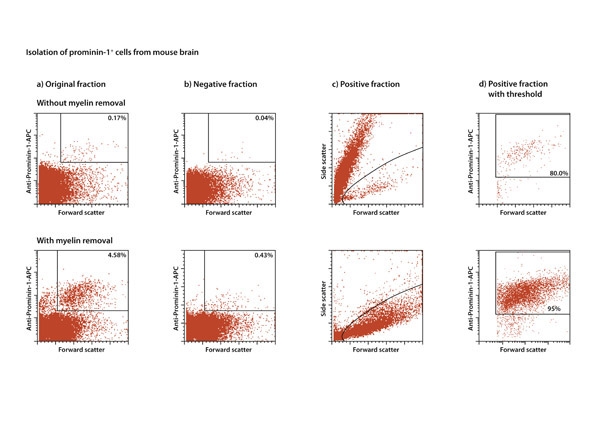
 **Figure. 3** Myelin debris in single-cell suspensions considerably impairs cell isolation, and removal of myelin debris by Myelin Removal Beads increases efficiency cell separations. For MACS Separations using Anti-Prominin-1 MicroBeads, P22 mouse brain was dissociated using the Neural Tissue Dissociation Kit (P). Cells from the single-cell suspension were either directly used for separation, or were submitted to myelin depletion using Myelin Removal Beads. Comparing the separation from samples without and with myelin removal, demonstrates that the purity is higher for samples with previous myelin removal.

## Disclosures

The authors are employees of Miltenyi Biotec GmbH, Germany.

## Discussion

The gentleMACS Dissociator facilitates the standardized preparation of single-cell suspensions from neural tissues in a closed system. Neural Tissue Dissociation Kits are optimized to preserve antigen epitopes needed for further applications like immunostainings and immunomagnetic cell separation^1-5^. In this protocol we show the gentle enzymatic dissociation of mouse brain using the gentleMACS Dissociator and the Neural Tissue Dissociation Kit (P), yielding in 97% viable cells. 100 mg of neural tissue yields between 5x10^6^ and 1x10^7^ cells, depending on the age of the host tissue. The targeted Prominin-1^+^ cells were isolated using Anti-Prominin-1 MicroBeads and subsequently taken into culture. The protocol is shown to be even more effective with the additional use of Myelin Removal Beads when working with neural tissue derived from mice >P7.
